# Surgical technique of temporal muscle resuspension during cranioplasty for minimizing temporal hollowing: A case series

**DOI:** 10.3389/fsurg.2022.996484

**Published:** 2022-09-23

**Authors:** Jingguo Yang, Xiaoyu Yang, Junjie Wang, Hang Yu, Chao You, Lu Ma, Junwen Guan

**Affiliations:** ^1^ Department of Neurosurgery, West China Hospital, Sichuan University, Chengdu, China; ^2^ Sichuan Provincial People’s Hospital, University of Electronic Technology, Chengdu, China

**Keywords:** cranioplasty, temporal hollowing, temporal muscle resuspension, case series, decompressive craniectomy

## Abstract

**Background:**

Temporal hollowing is a common but often overlooked complication following cranioplasty. To minimize temporal hollowing caused by temporal muscle contraction, we present the novel technique for temporal muscle resuspension during cranioplasty.

**Methods:**

This is a retrospective case series which were done by a single surgeon at our university tertiary-A hospital between January 2019 and February 2020. The surgical technique was performed according to the forms of temporal muscle based on preoperative 3-D reconstruction and intraoperative images. All patients were followed up and evaluated on esthetic and functional outcomes.

**Results:**

17 patients with an average age of 39.35 years, frontotemporoparietal cranial defect size of 78.85 cm^2^, and median follow-up of 7 months were included. The main cause of decompressive craniectomy was trauma (*n* = 15). Techniques of temporal muscle augmentative resuspension were performed. The follow-up esthetic and functional outcome evaluation showed that all patients had good postoperative results. No revision surgery was found among the patients.

**Conclusions:**

This study proposes methods of temporal muscle augmentative resuspension based on forms of the muscle. We believe this might be of use in minimizing temporal hollowing after cranioplasty.

## Introduction

Decompressive craniectomy (DC) is performed for the management of refractory intracranial pressure caused by head injury, ischemic, or hemorrhagic stroke (1). Secondary cranioplasty (CP) is an important procedure because it not only restores the structural integrity and protective barrier of the skull but also improves the neurological outcomes and prevents the Syndrome of the trephined (2–4). However, the post-surgery outcomes are not always satisfactory due to several reasons including the initial decompressive procedure and the secondary CP.

The management of the temporal muscle during the CP procedure is not much investigated. Many patients who have undergone a CP are left with temporal hollowing that troubles them both physically and psychologically (5). Although the etiologies remain unclear, the underlying mechanisms could be denervation and disruption of vascular and fat pads of the temporal muscle (6–8). Also, once the temporal muscle is detached from the bone during the initial surgery, its resuspension to the original anatomic position becomes challenging, and the retraction of the muscle results in temporal hollowing (9, 10).

Numerous techniques have been described to minimize the temporal hollowing and restore the symmetrical appearance in the patients. New materials used for customized CP and modified craniofacial reconstruction with 3-D techniques result in excellent cosmesis (3, 5, 11, 12). Techniques such as injection augmentation with autologous fat or dermal filler, native tissue augmentation, temporal muscle resuspension, use of alloplastic materials and new surgical techniques have been developed in correcting the soft tissue defects (13–18). Among these techniques, temporal muscle resuspension has been proven to be a key in restoring craniofacial symmetry (9, 10, 19). There are multiple methods of temporal muscle resuspension (9, 19–27). These methods use different techniques to anchor the muscle, preventing the hollowing after surgery. Unfortunately, the end results are not always satisfactory because temporal muscles of some patients undergo severe contraction, and the resuspension of such muscles is difficult (22). To deal with remaining temporal muscle individually, we proposed relevant disposals depending on forms of the muscle to minimize temporal deformity and lay foundations for future research.

## Methods

### Study design and patients

This retrospective case series study has been reported in line with the PROCESS guideline (28). The study protocol was supported by our university tertiary-A hospital ethical committee and was registered at Chinese Clinical Trial Registry (ChiCTR2200061822). Informed consents were obtained from all patients. All consecutive patients diagnosed with frontotemporoparietal cranial defects were included in our study. Those lost to follow up, follow-up time less than 6 months, or patients without intraoperative and postoperative photographs were excluded. Based on our experience, the original surgical technique research included 17 patients (7 female patients) who underwent CP between January 2019 and February 2020. A single multidisciplinary team performed CP in these patients, all patients operated by a senior surgeon in neurosurgery with wide experience in cranioplasty.

All patients were routinely followed up at 1, 3, 6, 12 months postoperatively in outpatient department. Demographic and clinical features included sex, age, cause of DC, defect size, internal between DC and CP, implant material and follow-up time. RFCC-score (29) is an objective, time-saving and reliable scoring system, which is applicable to nearly all the post-CP patients and is used to evaluate different surgical techniques and complications associated with CP. The score is based on four parameters: skin, fitting of cranioplasty, appearance symmetry and function. The final functional and esthetic results were evaluated using the RFCC-score by the patients themselves and the independent neurosurgeons. All the patients were able to communicate properly, express their experiences, and evaluate their cosmetic results.

### Classifications of the temporal muscle during CP

**Figure 1:**
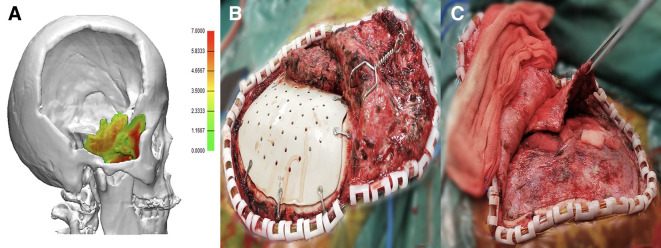
(**A**) Preoperative virtual 3-D temporal muscle reconstruction modeling. (**B**) Intraoperative hypertrophic temporal muscle: the muscle is retracted severely and performs hypertrophic. (**C**) The intraoperative image shows that the flat temporal muscle is thin and flat.

During the planning period, each patient received cranial spiral CT imaging, and virtual 3-D models for the temporal muscle were reconstructed. The remaining temporal muscle within the defect area was reconstructed using Mimics (Materialise, Belgium) and 3-matic (Materialise, Belgium) (Figure 1A), followed by the classifications of the temporal muscle. Then, we dissect the remaining temporal muscle and the thickness of the muscle was verified during cranioplasty. The flowchart of the temporal muscle reconstruction and classifications are shown in Supplementary Figures S1, S2.

#### Hypertrophic temporal muscle

The preoperative temporal muscle reconstruction image shows that the temporal muscle performs severely contracted with hypertrophic thickness and the form could be further confirmed intraoperatively (Figure 1B). Therefore, we define this kind of muscle as hypertrophic temporal muscle.

#### Flat temporal muscle

As per the preoperative reconstruction image, the temporal muscle was found to be thin within the defect region, and the muscle performed thin and flat intraoperatively (Figure 1C). So, we define this kind of muscle as flat temporal muscle.

### Surgical techniques

In the intraoperative procedure, we made a semilunar incision along the old scar and opened the flap. Firstly, skin and the subcutaneous layers were dissected to expose the dura and the fascia of the temporal muscle. Traditional scalper was used to dissect the skin flap. Secondly, the scalp flap was reflected and fixed with scalp hooks. After debriding the bone margins with a monopolar coagulator, we intensively dissected the temporal muscle using a scissors while preserving the integrity of the dura to the greatest extend. After dissecting the temporal muscle from the dura, the PEEK or titanium implant was placed under the muscle to avoid temporal depression. Finally, the temporal muscle was disposed according to the aforementioned muscle classifications. During the process, if the electrocautery was necessary, the power was limited relatively low to minimize the heat and electric stimulation. The disposal methods are described as follows:

#### Hypertrophic temporal muscle

**Figure 2:**
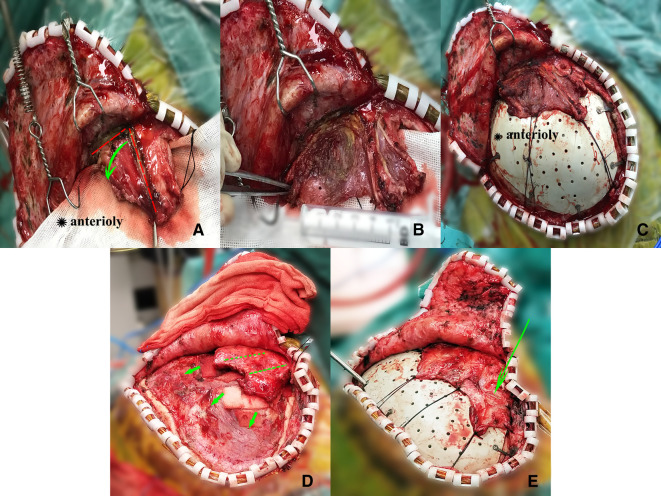
(**A–C**) An illustrative case. A 40-year-old female with frontotemporoparietal cranial defect due to brain trauma and her temporal muscle was hypertrophic. (**A**) The L-shaped incision (red dashed line) was made using monopolar coagulator with the depth deeper than the temporalis fascia and along the superficial layer of the muscle. The superficial muscle layer along with the fascia layer was dissected using the scissors and the green arrow indicates that the two layers were reflected anterosperiorly. (**B**) Reflection of the two layers of the temporalis anterosperiorly to mimic the muscle's original anatomy. (**C**) The temporal muscle is fixed onto the titanium mesh plates or the dissected periosteum using sutures to sustain its tension. (**D,E**) A 33-year-old male with cranial defect after intracranial hematoma and his temporal muscle performs flat. (**D**) The straight-line incisions made along the superficial temporal fascia. (**E**) The green arrow indicated that the incisions made along the fascial and the extended temporalis is fixed using the same way.

The augmentative method is illustrated in Figure 2. Intraoperatively, from the root of the temporal muscle, an L-shaped incision was made using the monopolar coagulator with the depth deeper than the temporal fascia and along the superficial layer of the muscle (Figure 2A). The superficial muscle layer along with the fascia layer was then dissected and reflected to mimic the muscle's original anatomy (Figure 2B). The direction of reflection depended on the position of the temporal muscle (Figure 2C, the muscle is reflected anteriorly) to restore its original anatomical form. Dissections were carried out with a monopolar coagulator, and bleeding was controlled with a bipolar coagulator. Finally, the implant plates acted as a fixation point onto which the augmentative temporal muscle can be tightly sutured and sustained its tension. In this way, we sufficiently extended the temporal muscle so it reaches its original anatomy as much as possible.

#### Flat temporal muscle

The surgical dissection procedure was the same as described earlier; however, for patients whose temporal muscles were thin and flat, making incisions along the muscles was very likely to damage the vessels and nerves. To make the temporal muscle reach its original anatomy to greatest extent possible, we made incisions along the superficial temporal fascia using scissors (Figure 2D). Thus, we could not only reduce the tension induced by the temporal fascia and extend the temporal muscle sufficiently (Figure 2E) but also make it less likely to damage the nerves and vessels. The temporal muscle was sutured as previously described.

## Results

The patient group consisted of 10 male patients and 7 female patients with an average age of 39.35 ± 3.72 years and a median follow-up of 7 months (range, 6–15 months). The causes of the defects were trauma (*n* = 15) and vascular issues (*n* = 2). The average defect size was 78.85 ± 4.62 cm^2^, and the average time between DC and CP was 7 months. There were also differences in the implant material: titanium implant (*n* = 4), PEEK implant (*n* = 12) and autologous bone (*n* = 1). According to preoperative 3-D reconstruction and intraoperative images: 6 patients were found to have hypertrophic temporal muscle, 11 had flat temporal muscle. Details of the patients are summarized in Table 1.

**Table 1:** Summary of patient demographics and classifications of the temporal muscle.

Patient	Sex	Age (y)	Cause of DC	Temporal muscle classification	Defect size (cm^2^)	Interval between craniectomy and cranioplasty (m)	Implant material	Follow-up time (m)
case1	M	50	Trauma	Hypertrophic	105.14	3	Titanium	14
case2	F	40	Trauma	Hypertrophic	65.36	8	PEEK	15
case3	F	77	Trauma	Hypertrophic	67.48	8	Titanium	6
case4	F	51	Trauma	Hypertrophic	62.22	20	Titanium	7
case5	M	30	Vascular	Hypertrophic	72.49	8	PEEK	8
case6	M	36	Trauma	Hypertrophic	103.5	4	PEEK	9
case7	M	53	Trauma	Flat	91.85	6	PEEK	6
case8	M	44	Trauma	Flat	51.4	9	PEEK	13
case9	F	48	Trauma	Flat	83.03	5	PEEK	6
case10	M	16	Trauma	Flat	60.27	5	PEEK	7
case11	M	27	Trauma	Flat	84.78	8	PEEK	6
case12	M	48	Trauma	Flat	77.02	3	PEEK	6
case13	F	39	Trauma	Flat	123.59	12	PEEK	6
case14	M	33	Vascular	Flat	61.12	7	PEEK	10
case15	F	36	Trauma	Flat	76.9	14	PEEK	9
case16	M	30	Trauma	Flat	66.43	4	Titanium	8
case17	F	11	Trauma	Flat	87.92	4	Autologous bone	6
Average	7F;10M	39.35			78.85	7	12 PEEK 4 Titanium 1 Autologous bone	7

DC, decompressive craniectomy; M, male; F, female; PEEK, polyetheretherketone.

**Figure 3:**
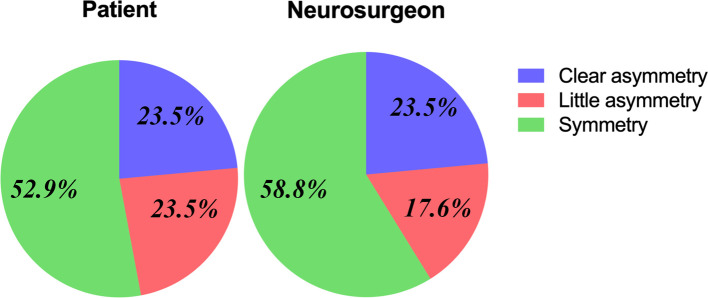
Evaluation of esthetic outcome from patients themselves and neurosurgeons and most patients show good esthetic results.

The RFCC-scores (29) evaluated by the patients and neurosurgeons showed the functional and esthetic outcomes of the patients. The surgeons and patients evaluated the mean scores as 5.5 and 5.2 respectively. These observations prove that the results are reliable (Table 2). 14 patients proved to be satisfactory (symmetrical or little asymmetry) in terms of appearance as per the RFCC-score (Figure 3). 4 out of 17 patients showed visible asymmetry. Among these four patients, three patients developed facial nerve injury, and two showed restricted jaw movement. No revision surgery was found among the patients.

**Table 2:** Patients RFCC-scores.

Reviewer	Mean	Median	Range
Patient	5.2	5	4–8
Surgeon	5.5	5	4–10

## Discussion

Most neurosurgeons concentrate solely on restoring the bone continuity, while neglecting the coexisting soft tissue change. Hence, the temporal hollowing becomes a problem. Nowadays, patients not only pay attention to the protective barrier or functional improvement after CP but also favor the esthetic outcomes. Some patients even choose multiple surgeries to correct the temporal deformity. This further increases the incidence of infection and poses economic burden to patients (30). Therefore, the post-CP temporal hollowing should be minimized as much as possible.

Post-CP inferior displacement of the temporal muscle can lead to temporal hollowing, and adequate resuspension during CP is paramount for the prevention of temporal hollowing (10). Although, conventional resuspension is common and several novel methods for temporal muscle resuspension exist (9, 19, 22–27), adequate coverage is not always possible if the muscle is severely contracted and difficult to restore to its original anatomical structure (22). Therefore, neurosurgeons should consider various forms of temporal muscle during the planning phase when the patient is suitable for CP. Through the computer-assisted technology, we have performed preoperative temporal muscle 3-D reconstruction and proposed surgical technique of temporal muscle augmentative resuspension based on preoperative 3-D reconstruction and intraoperative images.

In this research, the temporal muscle management focuses on muscle augmentation and resuspension. For patients with a hypertrophic temporal muscle, the muscle severely contracts, and the conventional temporal muscle resuspension is difficult; therefore, existing tissue expansion is necessary. Incisions are not routinely made in the muscle during the CP. However, the dissection between the muscle was carried out as previously described (12) and the anatomy of the muscle shows that the nerves and vessels travel superficially to the sub-periosteum (7, 31, 32). Therefore, making the incisions along the superficial layer could reduce the risk of damaging the nerves and vessels. For those patients whose temporal muscle is thin and flat, adequate resuspension of the muscle along its original anatomy is vital. A systematic review concluded that the superficial temporal fascia is not relevant to temporal hollowing (31); therefore, the incisions made along the layer could not only reduce the risk of damaging the vessels but also decrease the tension of the fascia. After that, the muscle can be fixed using sutures. After temporal muscle augmentation, the muscle would be sufficiently extended to reach closer to its original anatomy.

**Figure 4:**
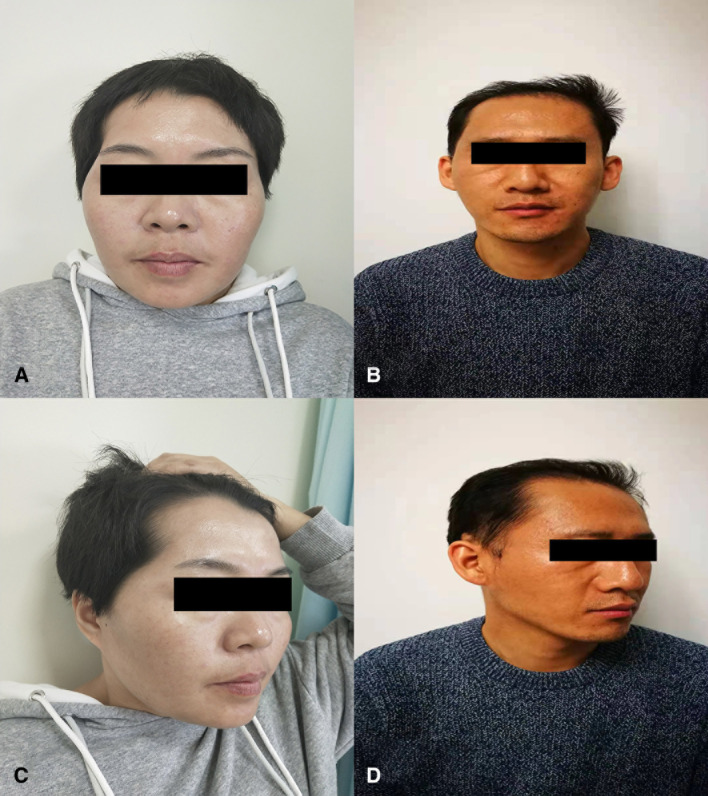
Postoperative photographs of the patient. (**A,C**), Patient with hypertrophic temporal muscle and postoperative anteroposterior and lateral views 15 months after temporalis management. (**B**,**D**), Patients with flat temporal muscle and postoperative anteroposterior and lateral views 10 months after surgery. The natural contour of their lateral orbital rim and lack of temporal deformity.

Previously, the various forms of the temporal muscle were less studied; however, these forms can affect muscle management and outcomes. This study uses a multidisciplinary approach to perform 3-D reconstruction of temporal muscle and propose an initial classification of the remaining temporal muscle. Also, the described management methods are simple and quick (takes only a couple of minutes of operation time). Moreover, our technique implements autologous tissues, so there is a decreased the incidence of infection. In comparison with the traditional techniques of temporal muscle suspension, our technique helps to dispose the temporal muscle according to different situations and restore the muscle's original anatomy as much as possible. We also examined the appearance of patients during the follow-up and further evaluated the esthetic and functional outcome. During the average 7 months of follow-up, the esthetic and functional outcome are good enough (Figure 4) which is comparable to the temporal hollowing rate of 52% in a previously reported study (10). Although our technique was not able to completely eliminate temporal hollowing, we were able to minimize the incidence of the complications and reduce the possible costs for multiple reconstructive surgeries (30). Therefore, our surgical techniques based on various forms of the temporal muscle could provide a new systematic approach for minimizing the temporal hollowing after surgery.

Our study has some limitations that require further consideration. The preliminary study mainly focused on novel technique of temporal muscle augmentation and resuspension. However, our hypothesis about the temporal muscle classification has not been proved yet and needs further investigation. Also, this is a case series and technical note study with a small sample size, this represents one weakness of our analysis. Another limitation is the evaluation of the esthetic outcome, since this assessment was relatively arbitrary. However, the RFCC score seems to be valid tool, enabling to evaluate the surgical technique as to its functional and esthetic outcome. Next, our technique requires the temporal muscle to be dissected from dura, even if the strong adhesion of the temporal muscle and dura carries some risk of CSF leakage and of damage to the brain. Therefore, careful attention is required during the muscle dissection. Besides, we have not analyzed the risk factors other than anatomic structure, that could result in temporal hollowing. This analysis will become object of our future research.

Despite the limitations, the long-term follow-ups were generally satisfying. Moreover, the temporal muscle augmentative resuspension technique will aid in expanding the current knowledge. We believe that our approaches might prove useful in minimizing the temporal hollowing after CP. Moving forward, patient-specific strategies and multidisciplinary efforts are needed to explore various types of implanted materials to camouflage the temporal deformity and tissue-engineered advancements in 3-D bioprinting.

## Conclusion

This is the first study to propose methods of temporal muscle augmentative resuspension based on forms of the muscle. These techniques are expected to minimize the temporal hollowing after cranioplasty. However, our findings about temporal muscle classification needs to be confirmed in future studies.

## Data Availability

The raw data supporting the conclusions of this article will be made available from the corresponding author upon reasonable request.
