# Supplementary material for: Surgical technique of temporal muscle resuspension during cranioplasty for minimizing temporal hollowing: A case series
Source: Front Surg. 2022 Sep 23;9:996484. doi: 10.3389/fsurg.2022.996484 (PMC9632970; doi:10.3389/fsurg.2022.996484)
Supplement: Supplementary file 2 [file Datasheet2.docx]

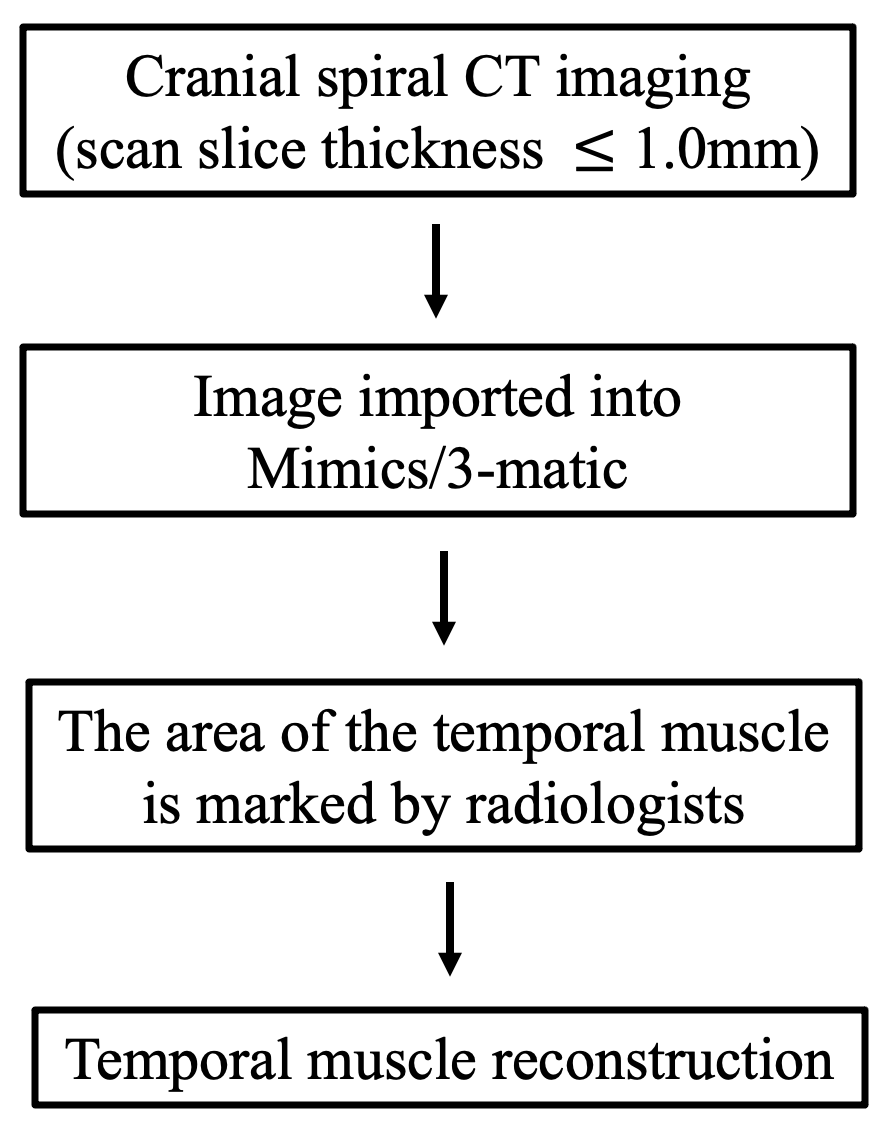


Figure S1. Flowchart of the temporal muscle reconstruction.


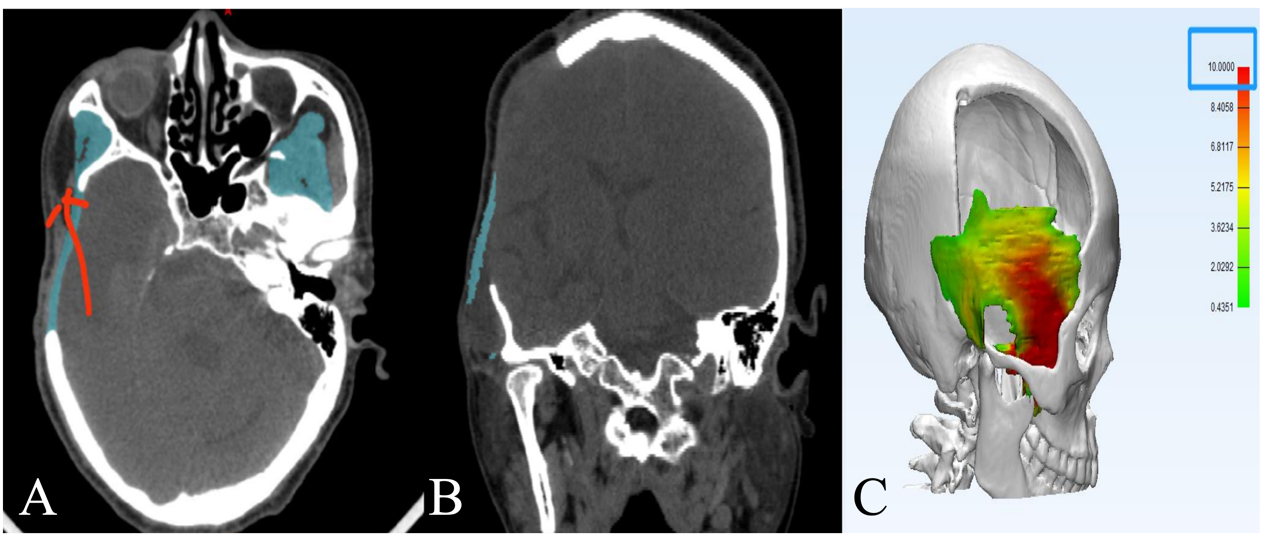


Figure S2. (A.B) The area of the temporal muscle is marked (light blue) by experienced radiologists using cranial spiral CT imaging. (C) The reconstruction image shows the area and thickness of the remaining temporal muscle.

Figure S1. Shows the flowchart of the temporal muscle reconstruction. During the planning period, each patient received cranial spiral CT imaging (scan less than 1mm slice thickness), the remaining area of the temporal muscle were marked by experienced radiologists (Fig S2.A.B) and reconstructed using Mimics and 3-matic. The reconstruction image of preoperative temporal muscle is shown in Fig 1.A and Fig S2.C and the reconstruction image presents the thickness of the remaining muscle. As the remaining area of the temporal muscle marked by radiologists is not always accurate. Therefore, we dissect the remaining temporal muscle and the thickness of the remaining temporal muscle was verified during cranioplasty.

Study shows [1] that the temporal muscle is thickest anteriorly (1.10-1.40 cm). In our experience, after we dissect the temporal muscle intraoperative, if the remaining temporal severely contracted and was hypertrophic with a marginal thickness over 1cm, we define this kind of muscle as hypertrophic temporal muscle (Fig.1 B). When the remaining temporal muscle looks thin and flat (with a thickness less than 1cm), we define this kind of muscle as flat temporal muscle (Fig.1 C). However, this study mainly focused on technique of temporal muscle augmentation and resuspension and the temporal muscle classifications need further verification.


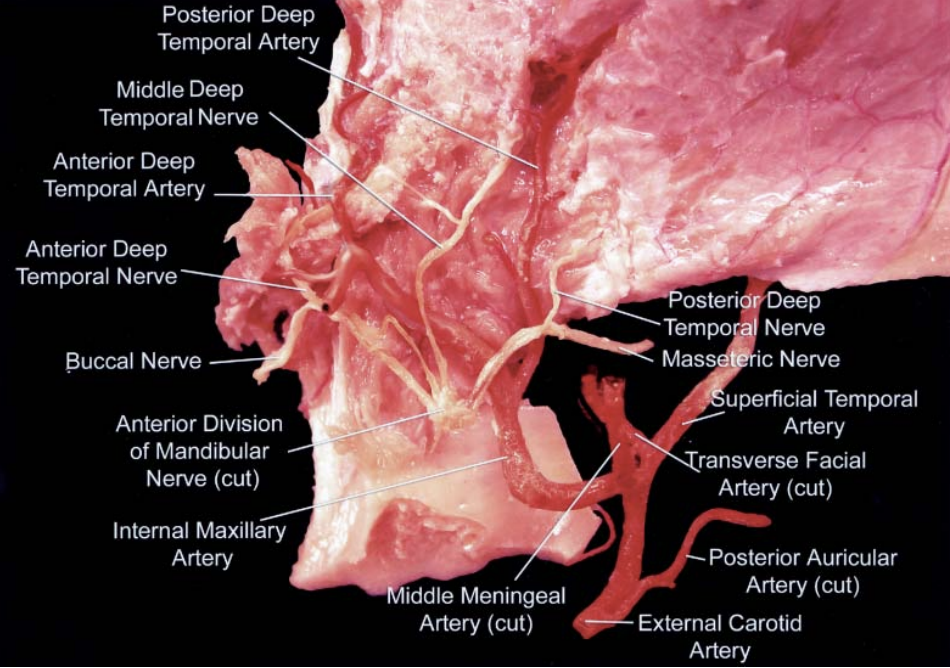


Figure S3. The anatomical basis of temporal muscle shows that the nerves and vessels travel superficially to the sub-periosteum. Reproduced with permission^2^. Copyright 2004, thejns.

1. Vaca EE, Purnell CA, Gosain AK, Alghoul MS. Postoperative temporal hollowing:Is there a surgical approach that prevents this complication? A systematic review and anatomic illustration. J Plast Reconstr Aesthet Surg. 2017 Mar;70(3):401-415.
2. Kadri PA, Al-Mefty O. The anatomical basis for surgical preservation of temporal muscle. J. Neurosurg. 2004; 100: 517-22.
